# Lectures on Bright’s Disease

**Published:** 1889-07

**Authors:** 


### BOOK NOTICES AND REVIEWS.

Lectures on Bright's Disease. By Robert Saundby, M. D. Edin., with fifty illustrations. New York: E. B. Treat, 771 Broadway. 1889. Price, $2.75.

This book of 290 pages gives the present state of knowledge upon Bright's Disease. It is derived from an experience of thirteen years, devoted by the author to the study of the subject of kidney changes.

The work is divided into three principal parts, or sections: I, General pathology; II, Clinical examination of the urine; III, Bright's Disease in its history, classification, etiology, pathology, complications, and treatment.

The author very strongly insists upon the unreliability of the occurrence of albumen in the urine as a diagnostic sign of Bright's. He gives the list of diseased conditions other than Bright's in which albumen occurs:

> Diseases of the heart, lungs, and liver; in peritonitis, pregnancy, abdominal tumors, in most febrile and inflammatory diseases, many cases of poisoning, in cancer, tubercle, and syphilis; in lardaceous disease. anemia, debility, dyspepsia. purpura, scurvy, after paroxysmal hæmoglobinuria, in gout, in delirium tremens, various diseases of brain and spinal cord, in epilepsy, some skin diseases, and in healthy persons after bathing, exercise, etc. [He might also have added after the administration of chloroform.] All these states may be arranged under the following groups: 1. Congestion of kidney * * * 2. Inflammation, acute or chronic, inflammatory, zymotic, and septic diseases, in gout, chronic lead poisoning, etc. 3. New growths, cancer, tubercle, or syphilitic deposits in the kidney. 4. Degenerations. 5. Alterations in the composition of the blood as in purpura, scurvy, etc.

He then adds: "Looked at in this way, the difficulties which have beset the discussion of the significance of albuminuria melt away; this result is attained by *the absolute surrender of the doctrine that albuminuria signifies Bright's disease*, and the acceptance of the view that it is simply the admixture of albumen derived from the blood serum with the urine." In another place he says: "Whatever may have been the case twenty years ago, it cannot be maintained now, and certainly will not be admitted here, that albuminuria and Bright's disease are synonymous." He asserts that many cases of Bright's disease go on to termination without the occurrence of albuminuria. What, then, does he depend upon for a constant diagnostic sign of Bright's? It is the occurrence of the three kinds of tube casts in the urine: (1) Blood casts; (2) Epithelial casts; (3) Hyaline casts.

In classifying the different varieties of Bright's disease, he abandons the arrangements of Virchow, Rosenstein, Grainger Stewart, etc., and gives the following divisions: (1) Febrile nephritis; (2) Toxæmic nephritis; (3) Obstructive nephritis.

"Lardaceous or waxy kidney is not made a special group because it is only when associated with chronic nephritis that it deserves to be called Bright's disease. * * * The lardaceous kidney of most authors is chronic nephritis as it occurs in chronic pyrexial diseases—*e. g.*, long-standing suppurations, phthisis, etc., in which lardaceous degeneration may occur just as it may in any form of chronic Bright's disease."

In chapter fourteenth the complications of chronic Bright's disease are dwelt upon and a table of them given. At the end of each chapter is given the bibliography of the subject treated in that chapter—an excellent arrangement for the convenience of those readers who wish to be fully instructed in all the authorities.

We can well recommend this book as giving, in a compact form, the latest views upon the subject of nephritis. Of course, the treatment advised will not particularly commend itself to a homœopathist, but that need not stand in the way of his getting a very excellent and clear idea of kidney diseases as at present understood. W. M. J.

